# Optical space-time wave packets having arbitrary group velocities in free space

**DOI:** 10.1038/s41467-019-08735-8

**Published:** 2019-02-25

**Authors:** H. Esat Kondakci, Ayman F. Abouraddy

**Affiliations:** 1 CREOL, The College of Optics & Photonics, University of Central Florida, Orlando, FL 32816 USA; 2 Department of Physics and Astronomy, Purdue University, West Lafayette, IN 47907 USA

## Abstract

Controlling the group velocity of an optical pulse typically requires traversing a material or structure whose dispersion is judiciously crafted. Alternatively, the group velocity can be modified in free space by spatially structuring the beam profile, but the realizable deviation from the speed of light in vacuum is small. Here we demonstrate precise and versatile control over the group velocity of a propagation-invariant optical wave packet in free space through sculpting its spatio-temporal spectrum. By jointly modulating the spatial and temporal degrees of freedom, arbitrary group velocities are unambiguously observed in free space above or below the speed of light in vacuum, whether in the forward direction propagating away from the source or even traveling backwards towards it.

## Abstract (web summary)

Controlling the group velocity of light in free space has been limited to small deviations so far. Here, the authors present a method to control the spatio-temporal spectrum and allow arbitrary group velocities of a wave packet in free space both above and below the speed of light.

## Introduction

The publication of Einstein’s seminal work on special relativity initiated an investigation of the speed of light in materials featuring strong chromatic dispersion^1^. Indeed, the group velocity *v*_g_ of an optical pulse in a resonant dispersive medium can deviate significantly from the speed of light in vacuum *c*, without posing a challenge to relativistic causality when *v*_g_ > *c* because the information speed never exceeds *c*^1,2^. Modifying the temporal spectrum in this manner is the basic premise for the development of the so-called slow light and fast light^3^ in a variety of material systems including ultracold atoms^4^, hot atomic vapors^5,6^, stimulated Brillouin scattering in optical fibers^7^, and active gain resonances^8,9^. Additionally, nanofabrication yields photonic systems that deliver similar control over the group velocity through structural dispersion in photonic crystals^10^, metamaterials^11^, tunneling junctions^12^, and nanophotonic structures^13^. In general, resonant systems have limited spectral bandwidths that can be exploited before pulse distortion obscures the targeted effect, with the pulse typically undergoing absorption, amplification, or temporal reshaping, but without necessarily affecting the field spatial profile.

In addition to temporal spectral modulation, it has been recently appreciated that structuring the spatial profile of a pulsed beam can impact its group velocity in free space^14–16^. In a manner similar to pulse propagation in a waveguide, the spatial spectrum of a structured pulsed beam comprises plane wave contributions tilted with respect to the propagation axis, which undergo larger delays between two planes than purely axially propagating modes. A large-area structured beam (narrow spatial spectrum) can travel for longer distances before beam deformation driven by diffraction and space-time coupling, but its group velocity deviates only slightly from *c*; whereas a narrow beam deviates further from *c*, but travels a shorter distance. Consequently, *v*_g_ is dependent on the size of the field spatial profile, and the maximum group delay observable is limited by the numerical aperture. Only velocities slightly lower than *c* (≈0.99999*c*) have been accessible in the experiments performed to date with maximum observed group delays of ~30fs, corresponding to a shift of ~10 μm over a distance of 1 m (or 1 part in 10^5^).

Another potential approach to controlling the group velocity of a pulsed beam in free space relies on sculpting the spatio-temporal profile of propagation-invariant wave packets^17,18^. Instead of manipulating separately the field spatial or temporal degrees of freedom and attempting to minimize unavoidable space-time coupling, tight spatio-temporal correlations are intentionally introduced into the wave packet spectrum, thereby resulting in the realization of arbitrary group velocities: superluminal, luminal, or subluminal, whether in the forward direction propagating away from the source or in the backward direction traveling toward it. The group velocity here is the speed of the wave packet central spatio-temporal intensity peak, whereas the wave packet energy remains spread over its entire spatio-temporal extent. Judiciously associating each wavelength in the pulse spectrum with a particular transverse spatial frequency traces out a conic section on the surface of the light-cone while maintaining a linear relationship between the axial component of the wave vector and frequency^19,20^. The slope of this linear relationship dictates the wave packet group velocity, and its linearity eliminates any additional dispersion terms. The resulting wave packets propagate free of diffraction and dispersion^17–22^, which makes them ideal candidates for unambiguously observing group velocities in free space that deviate substantially from *c*.

There have been previous efforts directed at producing optical wave packets endowed with spatio-temporal correlations. Several strategies have been implemented to date, which include exploiting the techniques associated with the generation of Bessel beams, such as the use of annular apertures in the focal plane of a spherical lens^23^ or axicons^24–26^, synthesis of X-waves^27^ during nonlinear processes such as second-harmonic generation^28^ or laser filamentation^29,30^, or through direct filtering of the requisite spatio-temporal spectrum^31,32^. The reported superluminal speeds achieved with these various approaches in free space have been to date 1.00022*c*^25^, 1.00012*c*^26^, 1.00015*c*^33^, and 1.111*c* in a plasma^24^. Reports on measured subluminal speeds have been lacking^17^ and limited to delays of hundreds of femtoseconds over a distance of 10 cm^34,35^, corresponding to a group velocity of ≈0.999*c*. There have been no experimental reports to date on negative group velocities in free space.

Here, we synthesize space-time (ST) wave packets^20,36,37^ using a phase-only spatial light modulator (SLM) that efficiently sculpts the field spatio-temporal spectrum and modifies the group velocity. The ST wave packets are synthesized for simplicity in the form of a light sheet that extends uniformly in one transverse dimension over ~25 mm, such that control over *v*_g_ is exercised in a macroscopic volume of space. We measure *v*_g_ in an interferometric arrangement utilizing a reference pulsed plane wave and confirm precise control over *v*_g_ from 30*c* in the forward direction to −4*c* in the backward direction. We observe group delays of ~±30 ps (three orders-of-magnitude larger than those in refs. ^14,15^), which is an order-of-magnitude longer than the pulse width, and is observed over a distance of only ~10 mm. Adding to the uniqueness of our approach, the achievable group velocity is independent of the beam size and of the pulse width. All that is needed to change the group velocity is a reorganization of the spectral correlations underlying the wave packet spatio-temporal structure. The novelty of our approach is its reliance on a linear system that utilizes a phase-only spatio-temporal Fourier synthesis strategy, which is energy efficient and precisely controllable^20^. Our approach allows for endowing the field with arbitrary, programmable spatio-temporal spectral correlations that can be tuned to produce—smoothly and continuously—any desired wave packet group velocity. The versatility and precision of this technique with respect to previous approaches is attested by the unprecedented range of control over the measured group velocity values over the subluminal, superluminal, and negative regimes in a single optical configuration. Crucially, while distinct theoretical proposals have been made previously for each range of the group velocity (e.g., subluminal^38–40^, superluminal^41^, and negative^42^ spans), our strategy is—to the best of our knowledge—the only experimental arrangement capable of controlling the group velocity continuously across all these regimes (with no moving parts) simply through the electronic implementation of a phase pattern imparted to a spectrally spread wave front impinging on a SLM.

## Results

### Concept of space-time wave packets

**Fig. 1:**
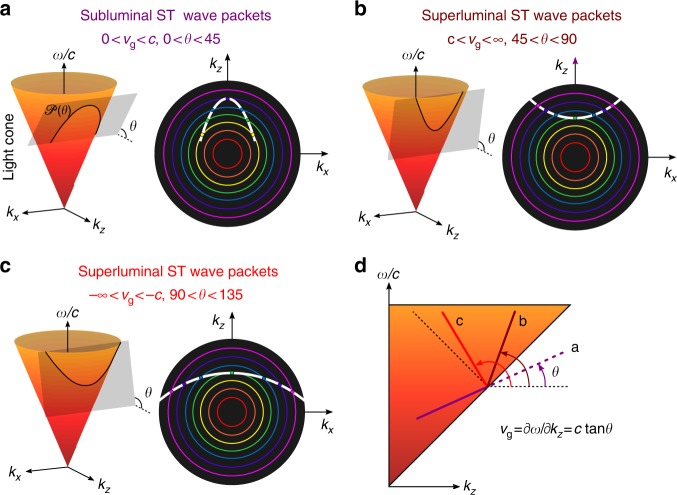
Spatio-temporal spectral engineering for arbitrary group velocity control. **a**–**c** Conic-section trajectories at the intersection of the free space light-cone with a spectral hyperplane ${\cal P}(\theta )$ are spatio-temporal spectral loci of space-time (ST) wave packets with tunable group velocities *v*_g_ in free space: **a** subluminal, **b** superluminal, and **c** negative-superluminal *v*_g_. For each case we plot the projection of the spatio-temporal spectrum onto the (*k*_*x*_, *k*_*z*_) plane restricted to *k*_*z*_ > 0 (white curve). **d** Projections of the spatio-temporal spectra from (**a**–**c**) onto the $(k_z,{\textstyle{\omega \over c}})$ plane. The slope of each projection determines the group velocity of each ST wave packet along the axial coordinate *z*, *v*_g_ = *c*tan*θ*

The properties of ST light sheets can be best understood by examining their representation in terms of monochromatic plane waves $e^{i(k_xx + k_zz - \omega t)}$, which are subject to the dispersion relationship $k_x^2 + k_z^2 = ({\textstyle{\omega \over c}})^2$ in free space; here, *k*_*x*_ and *k*_*z*_ are the transverse and longitudinal components of the wave vector along the *x* and *z* coordinates, respectively, *ω* is the temporal frequency, and the field is uniform along *y*. This relationship corresponds geometrically in the spectral space $(k_x,k_z,{\textstyle{\omega \over c}})$ to the surface of the light-cone (Fig. 1). The spatio-temporal spectrum of any physically realizable optical field compatible with causal excitation must lie on the surface of the light-cone with the added restriction *k*_*z*_ > 0. For example, the spatial spectra of monochromatic beams lie along the circle at the intersection of the light-cone with a horizontal iso-frequency plane, whereas the spatio-temporal spectrum of a traditional pulsed beam occupies a two-dimensional (2D) patch on the light-cone surface.

The spectra of ST wave packets do not occupy a 2D patch, but instead lie along a curved one-dimensional trajectory resulting from the intersection of the light-cone with a tilted spectral hyperplane ${\cal P}(\theta )$ described by the equation ${\textstyle{\omega \over c}} = k_{\mathrm{o}} + (k_z - k_{\mathrm{o}}){\mathrm{tan}}\theta$, where $k_{\mathrm{o}} = {\textstyle{{\omega _{\mathrm{o}}} \over c}}$ is a fixed wave number^20^, and the ST wave packet thus takes the form

1 $$\begin{array}{*{20}{l}} {E(x,z;t)} \hfill & = \hfill & {e^{i(k_{\mathrm{o}}z - \omega _{\mathrm{o}}t)}{\int} z{\mathrm{d}}k_x\tilde \psi (k_x)e^{i(k_xx + [k_z - k_{\mathrm{o}}][z - ct\,{\mathrm{tan}}\,\theta ])}} \hfill \\ {} \hfill & = \hfill & {e^{i(k_{\mathrm{o}}z - \omega _{\mathrm{o}}t)}\psi (x,z - v_{\mathrm{g}}t).} \hfill \end{array}$$

Therefore, the group velocity along the *z*-axis is $v_{\mathrm{g}} = {\textstyle{{\partial \omega } \over {\partial k_z}}} = c\,{\mathrm{tan}}\,\theta$, and is determined solely by the tilt of the hyperplane ${\cal P}(\theta )$. In the range 0 < *θ* < 45°, we have a subluminal wave packet *v*_g_ < *c*, and ${\cal P}(\theta )$ intersects with the light-cone in an ellipse (Fig. 1a). In the range 45° < *θ* < 90°, we have a superluminal wave packet *v*_g_ > *c*, and ${\cal P}(\theta )$ intersects with the light-cone in a hyperbola (Fig. 1b). Further increasing *θ* reverses the sign of *v*_g_ such that the wave packet travels backwards towards the source *v*_g_ < 0 in the range 90° < *θ* < 180° (Fig. 1c). These various scenarios are summarized in Fig. 1d.

### Experimental realization

**Fig. 2:**
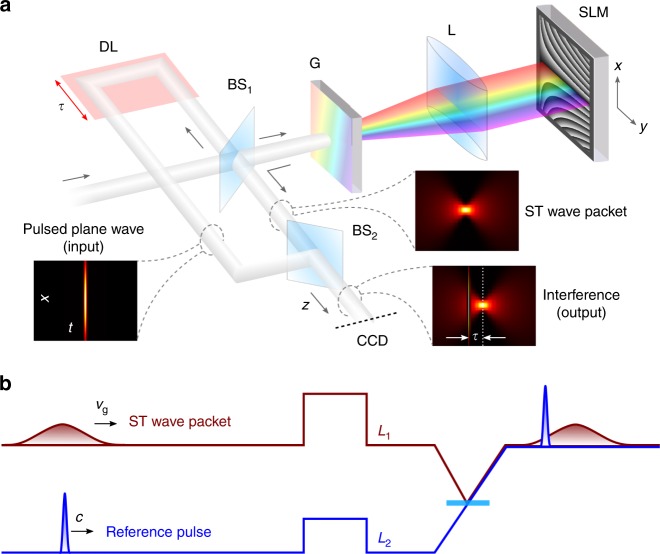
Synthesizing space-time (ST) wave packets and measuring their group velocity. **a** A pulsed plane wave is split into two paths: in one path the ST wave packet is synthesized using a two-dimensional pulse shaper formed of a diffraction grating (G), cylindrical lens (L), and spatial light modulator (SLM), while the other path is the reference. BS beam splitter, CCD charge-coupled device, DL delay line. The insets provide the spatio-temporal profile of a ST wave packet with a Gaussian spectrum, the reference pulsed plane wave, and their interference. See Methods and Supplementary Fig. 1 for details. **b** The reference and the ST wave packets are superposed, and the shorter reference pulse probes a fraction of the longer ST wave packet. Maximal interference visibility is observed when the selected delays *L*_1_ and *L*_2_ cause their peaks to coincide; see Methods and Supplementary Figs 2,3

**Fig. 3:**
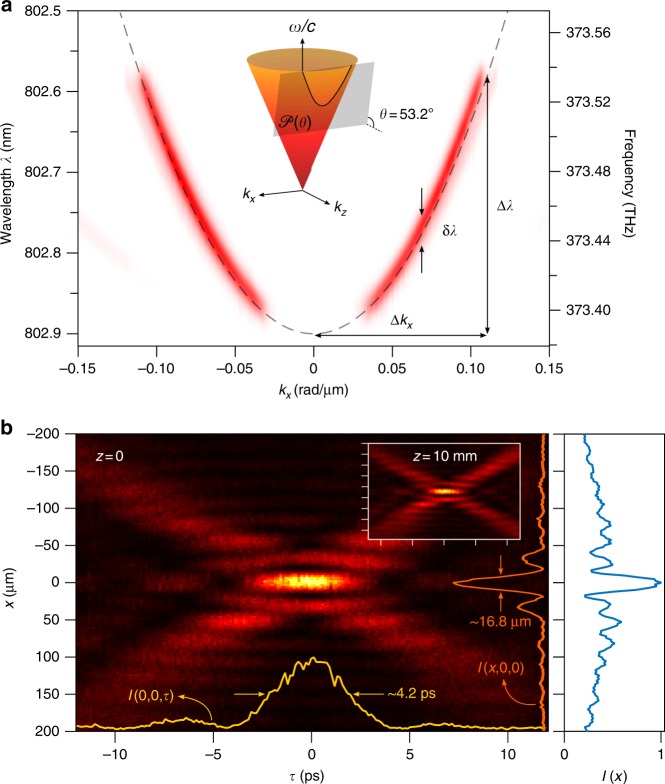
Spatio-temporal measurements of space-time (ST) wave packets. **a**, **b** Spatio-temporal **a** spectrum $|\tilde E(k_x,\lambda )|^2$ and **b** intensity profile *I*(*x*, 0, *τ*) for a ST wave packet with superluminal group velocity *v*_g_ = 1.34*c*, corresponding to a spectral hyperplane with *θ* = 53.2°. **b** The yellow and orange lines depict the pulse profile at *x* = 0, *I*(0, 0, *τ*), and beam profile at *τ* = 0, *I*(*x*, 0, 0), respectively. The inset shows the spatio-temporal intensity profile after propagating for *z* = 10 mm confirming the self-similar evolution of the ST wave packet. On the right, the normalized time-integrated beam profile $I(x,0) = {\int} {\mathrm{d}}\tau I(x,0,\tau )$ is given

We synthesize the ST wave packets by sculpting the spatio-temporal spectrum in the $(k_x,{\textstyle{\omega \over c}})$ plane via a 2D pulse shaper^20,43^. Starting with a generic pulsed plane wave, the spectrum is spread in space via a diffraction grating before impinging on a SLM, such that each wavelength *λ* occupies a column of the SLM that imparts a linear phase corresponding to a pair of spatial frequencies ±*k*_*x*_ that are to be assigned to that particular wavelength, as illustrated in Fig. 2a; see Methods. The retro-reflected wave front returns to the diffraction grating that superposes the wavelengths to reconstitute the pulse and produce the propagation-invariant ST wave packet corresponding to the desired hyperplane ${\cal P}(\theta )$. Using this approach we have synthesized and confirmed the spatio-temporal spectra of 11 different ST wave packets in the range 0° < *θ* < 180° extending from the subluminal to superluminal regimes. Figure 3a shows the measured spatio-temporal spectral intensity $|\tilde E(k_x,\lambda )|^2$ for a ST wave packet having *θ* = 53.2° and thus lying on a hyperbolic curve on the light-cone corresponding to a positive superluminal group velocity of *v*_g_ = 1.34*c*. The spatial bandwidth is Δ*k*_*x*_ = 0.11 rad/μm, and the the temporal bandwidth is Δ*λ* ≈ 0.3 nm. This spectrum is obtained by carrying out an optical Fourier transform along *x* to reveal the spatial spectrum. Our spatio-temporal synthesis strategy is distinct from previous approaches that make use of Bessel-beam-generation techniques and similar methodologies^23–26,41^, nonlinear processes^28–30^, or spatio-temporal filtering^31,32^. The latter approach utilizes a diffraction grating to spread the spectrum in space, a Fourier spatial filter then carves out the requisite spatio-temporal spectrum, resulting in either low throughput or high spectral uncertainty. In contrast, our strategy exploits a phase-only modulation scheme that is thus energy-efficient and can smoothly and continuously (within the precision of the SLM) tune the spatio-temporal correlations electronically with no moving parts, resulting in a corresponding controllable variation in the group velocity.

To map out the spatio-temporal profile of the ST wave packet *I*(*x*, *z*, *t*) = |*E*(*x*, *z*, *t*)|^2^, we make use of the interferometric arrangement illustrated in Fig. 2a. The initial pulsed plane wave (pulse width ~100 fs) is used as a reference and travels along a delay line that contains a spatial filter to ensure a flat wave front (see Methods and Supplementary Fig. 1). Superposing the shorter reference pulse and the synthesized ST wave packet (Eq. 1) produces spatially resolved interference fringes when they overlap in space and time—whose visibility reveals the spatio-temporal pulse profile (Fig. 2a and Supplementary Fig. 2). The measured intensity profile *I*(*x*, 0, *τ*) = |*E*(*x*, 0, *τ*)|^2^ of the ST wave packet having *θ* = 53.2° is plotted in Fig. 3b; *τ* is the delay in the reference arm. Plotted also are the pulse profile at the beam center *I*(0, 0, *τ*) = |*E*(*x* = 0, 0, *τ*)|^2^ whose width is ≈4.2 ps, and the beam profile at the pulse center *I*(*x*, 0, 0) = |*E*(*x*, 0, *τ* = 0)|^2^ whose width is ≈16.8 μm. Previous approaches for mapping out the spatio-temporal profile of propagation-invariant wave packets have made use of strategies ranging from spatially resolved ultrafast pulse measurement techniques^26,34,35^ to self-referenced interferometry^31^.

### Controlling the group velocity of a space-time wave packet

We now proceed to make use of this interferometric arrangement to determine *v*_g_ of the ST wave packets as we vary the spectral tilt angle *θ*. The setup enables synchronizing the ST wave packet with the luminal reference pulse while also uncovering any dispersion or reshaping in the ST wave packet with propagation. We first synchronize the ST wave packet with the reference pulse and take the central peak of the ST wave packet as the reference point in space and time for the subsequent measurements. An additional propagation distance *L*_1_ is introduced into the path of the ST wave packet, corresponding to a group delay of *τ*_ST_ = *L*_1_/*v*_g_≫Δ*τ* that is sufficient to eliminate any interference. We then determine the requisite distance *L*_2_ to be inserted into the path of the reference pulse to produce a group delay *τ*_r_ = *L*_2_/*c* and regain the maximum interference visibility, which signifies that *τ*_ST_ = *τ*_r_. The ratio of the distances *L*_1_ and *L*_2_ provides the ratio of the group velocity to the speed of light in vacuum *L*_1_/*L*_2_ = *v*_g_/*c*.

In the subluminal case *v*_g_ < *c*, we expect *L*_1_ < *L*_2_; that is, the extra distance introduced into the path of the reference traveling at *c* is larger than that placed in the path of the slower ST wave packet. In the superluminal case *v*_g_ > *c*, we have *L*_1_ > *L*_2_ for similar reasons. When considering ST wave packets having negative-*v*_g_, inserting a delay *L*_1_ in its path requires reducing the initial length of the reference path by a distance −*L*_2_ preceding the initial reference point, signifying that the ST wave packet is traveling backwards towards the source. As an illustration, the inset in Fig. 3b plots the same ST wave packet shown in the main panel of Fig. 3b observed after propagating a distance of *L*_1_ = 10 mm, which highlights the self-similarity of its free evolution^20^. The time axis is shifted by *τ*_r_ ≈ 24.88 ps, corresponding to *v*_g_ = (1.36 ± 4 × 10^−4^)*c*, which is in good agreement with the expected value of *v*_g_ = 1.34*c*.

**Fig. 4:**
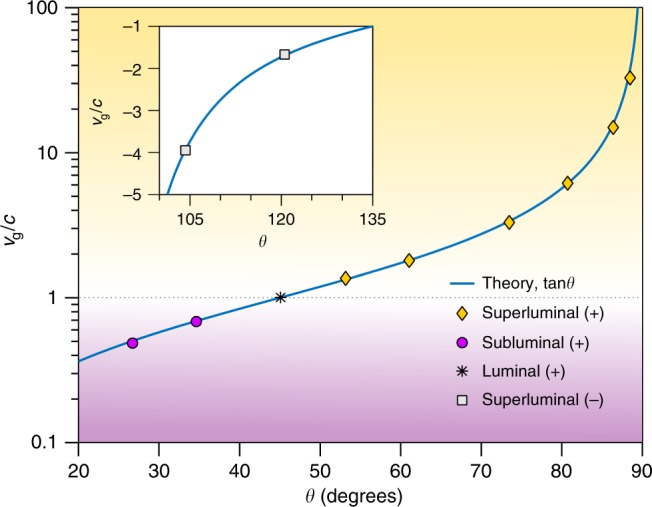
Measured group velocities for space-time (ST) wave packets. By changing the tilt angle *θ* of the spectral hyperplane ${\cal P}(\theta)$, we control *v*_g_ of the synthesized ST wave packets in the positive subluminal and superluminal regimes (corresponding to 0 < *θ* < 90°). We plot *v*_g_ on a logarithmic scale. Measurements of ST wave packets with negative-*v*_g_ (corresponding to *θ* > 90°) are given as points in the inset on a linear scale. The data in the main panel and in the inset are represented by points and both curves are the theoretical expectation *v*_g_ = *c*tan*θ*. The error bars are obtained from the standard error in the slope resulting from linear regression fits (see Methods and Supplementary Fig. 4). The error bars for the measurements are too small to appear, and are provided in Supplementary Table 1

The results of measuring *v*_g_ while varying *θ* for the positive-*v*_g_ ST wave packets are plotted in Fig. 4 (see also Supplementary Table 1). The values of *v*_g_ range from subluminal values of 0.5*c* to the superluminal values extending up to 32*c* (corresponding to values of *θ* in the range 0° < *θ* < 90°). The case of a luminal ST wave packet corresponds trivially to a pulsed plane wave generated by idling the SLM. The data are in excellent agreement with the theoretical prediction of *v*_g_ = *c*tan*θ*. The measurements of negative-*v*_g_ (90° < *θ* < 180°) are plotted in Fig. 4, inset, down to *v*_g_ ≈ −4*c*, and once again are in excellent agreement with the expectation of *v*_g_ = *c*tan*θ*.

## Discussion

An alternative understanding of these results makes use of the fact that tilting the plane ${\cal P}(\theta )$ from an initial position of ${\cal P}(0)$ corresponds to the action of a Lorentz boost associated with an observer moving at a relativistic speed of *v*_g_ = *c*tan*θ* with respect to a monochromatic source (*θ* = 0)^21,22,43^. Such an observer perceives in lieu of the diverging monochromatic beam a non-diverging wave packet of group velocity *v*_g_^44^. Indeed, at *θ* = 90° a condition known as time diffraction is realized where the axial coordinate *z* is replaced with time *t*, and the usual axial dynamics is displayed in time instead^21,43,45,46^. In that regard, our reported results here on controlling *v*_g_ of ST wave packets is an example of relativistic optical transformations implemented in a laboratory through spatio-temporal spectral engineering.

Note that it is not possible in any finite configuration to achieve a delta-function correlation between each spatial frequency *k*_*x*_ and wavelength *λ*; instead, there is always a finite spectral uncertainty *δλ* in this association. In our experiment, *δλ* ~ 24 pm (Fig. 3a), which sets a limit on the diffraction-free propagation distance over which the modified group velocity can be observed^36^. The maximum group delay achieved and the propagation-invariant length are essentially dictated by the spectral uncertainty *δλ* (see ref. ^36^ for details). The finite system aperture ultimately sets the lower bound on the value of *δλ*. For example, the size of the diffraction grating determines its spectral resolving power, the finite pixel size of the SLM further sets a lower bound on the precision of association between the spatial and temporal frequencies, whereas the size of the SLM active area determines the maximum temporal bandwidth that can be exploited. Of course, the spectral tilt angle *θ* determines the proportionality between the spatial and temporal bandwidths Δ*k*_*x*_ and Δ*λ*, respectively, which then links these limits to the transverse beam width. However, ST wave packets having the same spatial transverse width will propagate for different distances depending on the spectral uncertainty associated with each. The confluence of all these factors determine the maximum propagation-invariant distance and hence the maximum achievable group delays. Careful design of the experimental parameters helps extend the propagation distance^47^, and exploiting a phase plate in lieu of a SLM can extend the propagation distance even further^48^. Note that we have synthesized here optical wave packets where light has been localized along one transverse dimension but remains extended in the other transverse dimension. Localizing the wave packet along both transverse dimensions would require an additional SLM to extend the spatio-temporal modulation scheme into the second transverse dimension that we have not exploited here.

Finally, another strategy that also relies on spatio-temporal structuring of the optical field has been recently proposed theoretically^49^ and demonstrated experimentally^50^ that makes use of a so-called flying focus, whereupon a chirped pulse is focused with a lens having chromatic aberrations such that different spectral slices traverse the focal volume of the lens at a controllable speed, which was estimated by means of a streak camera.

We have considered here ST wave packets whose spatio-temporal spectral projection onto the $(k_z,{\textstyle{\omega \over c}})$ plane is a line. A plethora of alternative curved projections may be readily implemented to explore different wave packet propagation dynamics and to accommodate the properties of material systems in which the ST wave packet travels. Our results pave the way to novel schemes for phase matching in nonlinear optical processes^51,52^, new types of laser-plasma interactions^53,54^, and photon-dressing of electronic quasiparticles^55^.

## Methods

### Determining conic sections for the spatio-temporal spectra

The intersection of the light-cone $k_x^2 + k_z^2 = ({\textstyle{\omega \over c}})^2$ with the spectral hyperplane ${\cal P}(\theta )$ described by the equation ${\textstyle{\omega \over c}} = k_{\mathrm{o}} + (k_z - k_{\mathrm{o}})\,{\mathrm{tan}}\,\theta$ is a conic section: an ellipse (0° < *θ* < 45° or 135° < *θ* < 180°), a tangential line (*θ* = 45°), a hyperbola (45° < *θ* < 135°), or a parabola (*θ* = 135°). In all cases *v*_g_ = *c*tan*θ*. The projection onto the $(k_x,{\textstyle{\omega \over c}})$ plane, which the basis for our experimental synthesis procedure, is in all cases a conic section given by

2 $$\frac{1}{{k_1^2}}\left( {{\textstyle{\omega \over c}}{\kern 1pt} {\kern 1pt} \pm {\kern 1pt} {\kern 1pt} k_2} \right)^2 \pm \frac{{k_x^2}}{{k_3^2}} = 1,$$

where *k*_1_, *k*_2_, and *k*_3_ are positive-valued constants: ${\textstyle{{k_1} \over {k_{\mathrm{o}}}}} = \left| {{\textstyle{{{\mathrm{tan}}\theta } \over {1 + {\mathrm{tan}}\theta }}}} \right|$, ${\textstyle{{k_2} \over {k_{\mathrm{o}}}}} = {\textstyle{1 \over {|1 + {\mathrm{tan}}\theta |}}}$, and ${\textstyle{{k_3} \over {k_{\mathrm{o}}}}} = \sqrt {|{\textstyle{{1 + {\mathrm{tan}}\theta } \over {1 - {\mathrm{tan}}\theta }}}|}$. The signs in the equation are (−, +) in the range 0 < *θ* < 45° (an ellipse), (−,−) in the range 45° < *θ* < 90°, and (+, −) in the range 90° < *θ* < 135°.

In the paraxial limit where $k_x^{{\mathrm{max}}} \ll k_{\mathrm{o}}$, the conic section in the vicinity of *k*_*x*_ = 0 can be approximated by a section of a parabola,

3 $$\frac{\omega }{{\omega _{\mathrm{o}}}} = 1 + f(\theta )\frac{{k_x^2}}{{2k_{\mathrm{o}}^2}},$$

whose curvature is determined by *θ* through the function *f*(*θ*) given by

4 $$f(\theta ) = \frac{{{\mathrm{tan}}\,\theta }}{{{\mathrm{tan}}\,\theta - 1}}.$$

### Spatially resolved interference to obtain intensity profiles

We take the ST wave packet to be $E(x,z,t) = e^{i(k_{\mathrm{o}}z - \omega _{\mathrm{o}}t)}\psi (x,z - v_{\mathrm{g}}t)$ as provided in Eq. (1), and that of the reference plane wave pulse to be $E_{\mathrm{r}} = e^{i(k_{\mathrm{o}}z - \omega _{\mathrm{o}}t)}\psi _{\mathrm{r}}(z - ct)$. We have dropped the *x*-dependence of the reference and *ψ*_r_(*z*) is a slowly varying envelope. Superposing the two fields in the interferometer after delaying the reference by *τ* results in a new field ∝*E*(*x*, *z*, *t*) + *E*_r_(*x*, *z*, *t* − *τ*), whose time-average *I*(*x*, *τ*) is recorded at the output,

5 $$I(x,\tau ) \propto {\int} {\mathrm{d}}t\left| {E(x,z,t) + E_{\mathrm{r}}(x,z,t - \tau )} \right|^2.$$

We make use of the following representations of the fields for the ST wave packet and the reference pulse:

6 $$\begin{array}{*{20}{l}} {E(x,z,t)} \hfill & = \hfill & {e^{i(k_{\mathrm{o}}z - \omega _{\mathrm{o}}t)}{\int} {\mathrm{d}}k_x\tilde \psi (k_x)e^{ik_xx}e^{ - i(\omega - \omega _{\mathrm{o}})(t - z/v_{\mathrm{g}})}} \hfill \\ {} \hfill & = \hfill & {e^{i(k_{\mathrm{o}}z - \omega _{\mathrm{o}}t)}\psi \left( {x,t - z/v_{\mathrm{g}}} \right),} \hfill \end{array}$$

7 $$\begin{array}{*{20}{l}} {E_{\mathrm{r}}(x,z,t)} \hfill & = \hfill & {e^{i(k_{\mathrm{o}}z - \omega _{\mathrm{o}}t)}{\int} {\mathrm{d}}\omega \tilde \psi _{\mathrm{r}}(\omega - \omega _{\mathrm{o}})e^{i(\omega - \omega _{\mathrm{o}})(t - z/c)}} \hfill \\ {} \hfill & = \hfill & {e^{i(k_{\mathrm{o}}z - \omega _{\mathrm{o}}t)}\psi _{\mathrm{r}}\left( {x,t - z/c} \right).} \hfill \end{array}$$

We set the plane of the detector at *z* = 0 (CCD_1_ in our experiment; see Supplementary Fig. 1), from which we obtain the spatio-temporal interferogram

8 $$I(x,\tau ) \propto I_{{\mathrm{ST}}}(x) + I_{\mathrm{r}} + 2|R(x,\tau )|{\mathrm{cos}}(\omega _{\mathrm{o}}\tau - \varphi _{\mathrm{R}}(x,\tau )),$$

where

9 $$I_{{\mathrm{ST}}}(x) = {\int} {\mathrm{d}}t\left| {\psi (x,t)} \right|^2 = {\int} {\mathrm{d}}k_x\left| {\tilde \psi (k_x)} \right|^2(1 + {\mathrm{cos}}2k_xx),$$

10 $$I_{\mathrm{r}} = {\int} {\mathrm{d}}t\left| {\psi _{\mathrm{r}}(t)} \right|^2 = {\int} {\mathrm{d}}\omega \left| {\tilde \psi (\omega )} \right|^2,$$

where we have made the simplifying assumption that the spatial spectrum of the ST wave packet is an even function, $\tilde \psi (k_x) = \tilde \psi ( - k_x)$. This assumption is applicable to our experiment and does not result in any loss of generality. Note that *I*_ST_(*x*) corresponds to the time-averaged transverse spatial intensity profile of the ST wave packet, as would be registered by a charge-coupled device (CCD), for example, in the absence of an interferometer. Similarly, *I*_r_ is equal to the time-averaged reference pulse and represents constant background term. Note that *z* could be set at an arbitrary value because both the reference pulse and the ST wave packet are propagation invariant.

The cross-correlation function $R(x,\tau ) = |R(x,\tau )|e^{i\varphi _{\mathrm{R}}(x,\tau )}$ is given by

11 $$R(x,\tau ) = {\int} {\mathrm{d}}t\,\psi (x,t)\psi _{\mathrm{r}}^ \ast (t - \tau ).$$

Taking the integral over time *t* produces

12 $$R(x,\tau ) = {\int} {\mathrm{d}}k_x\tilde \psi (k_x)\tilde \psi _{\mathrm{r}}^ \ast (\omega )e^{ik_xx}e^{i(\omega - \omega _{\mathrm{r}})\tau },$$

where *ω* is no longer an independent variable, but is correlate to the spatial frequency *k*_*x*_ through the spatio-temporal curve at the intersection of the light-cone with the hyperspectral plane ${\cal P}(\theta )$. Because the reference pulse is significantly shorter that the ST wave packet, the spectral width of $\tilde \psi _{\mathrm{r}}$ is larger than that of $\tilde \psi$, so that one can ignore it, while retaining its amplitude,

13 $$R(x,\tau ) \approx \left| {\tilde \psi _{\mathrm{r}}(\omega _{\mathrm{o}})} \right|{\int} {\mathrm{d}}k_x\tilde \psi (k_x)e^{ik_xx}e^{i(\omega - \omega _{\mathrm{o}})\tau } = \left| {\tilde \psi _{\mathrm{r}}(\omega _{\mathrm{o}})} \right|\psi (x,\tau ).$$

Note that the spectral function $\tilde \psi (k_x)$ of the ST wave packet determines the coherence length of the observed spatio-temporal interferogram, which we thus expect to be on the order of the temporal width of the ST wave packet itself.

The visibility of the spatially resolved interference fringes (Supplementary Fig. 2) is given by

14 $$\nu (x,\tau ) = \frac{{2|R(x,\tau )|}}{{I_{ST}(x) + I_{\mathrm{r}}}}.$$

The squared visibility is then given by

15 $$\nu ^2(x,\tau ) \approx \frac{{4\left| {\tilde \psi _{\mathrm{r}}(\omega _{\mathrm{o}})} \right|^2\left| {\psi (x,\tau )} \right|^2}}{({I_{ST}(x) + I_{\mathrm{r}}})^{2}} \propto \left| {\psi (x,\tau )} \right|^2,$$

where the last approximation requires that we can ignore *I*_ST_(*x*) with respect to the constant background term *I*_r_ stemming from the reference pulse.

### Synthesis of ST wave packets

The input pulsed plane wave is produced by expanding the horizontally polarized pulses from a Ti:sapphire laser (Tsunami, Spectra Physics) having a bandwidth of ~8.5 nm centered on a wavelength of 800 nm, corresponding to pulses having a width of ~100 fs. A diffraction grating having a ruling of 1200 lines/mm and area 25 × 25 mm^2^ in reflection mode (Newport 10HG1200-800-1) is used to spread the pulse spectrum in space and the second diffraction order is selected to increase the spectral resolving power, resulting in an estimated spectral uncertainty of *δλ* ≈ 24 pm. After spreading the full spectral bandwidth of the pulse in space, the width size of the SLM (≈16 mm) acts as a spectral filter, thus reducing the bandwidth of the ST wave packet below the initial available bandwidth and minimizing the impact of any residual chirping in the input pulse. An aperture A can be used to further reduce the temporal bandwidth when needed. The spectrum is collimated using a cylindrical lens *L*_1−*y*_ of focal length *f* = 50 cm in a 2*f* configuration before impinging on the SLM. The SLM imparts a 2D phase modulation to the wave front that introduces controllable spatio-temporal spectral correlations. The retro-reflected wave from is then directed through the lens *L*_1−*y*_ back to the grating G, whereupon the ST wave packet is formed once the temporal/spatial frequencies are superposed; see Supplementary Fig. 1. Details of the synthesis procedure are described elsewhere^20,43,47,48^.

### Spectral analysis of ST wave packets

To obtain the spatio-temporal spectrum $|\tilde E(k_x,\lambda )|^2$ plotted in Fig. 3a in the main text, we place a beam splitter BS_2_ within the ST synthesis system to sample a portion of the field retro-reflected from the SLM after passing through the lens L_1−*y*_. The field is directed through a spherical lens L_4−s_ of focal length *f* = 7.5 cm to a CCD camera (CCD_2_); see Supplementary Fig. 1. The distances are selected such that the field from the SLM undergoes a 4*f* configuration along the direction of the spread spectrum (such that the wavelengths remain separated at the plane of CCD_2_), while undergoing a 2*f* system along the orthogonal direction, thus mapping each spatial frequency *k*_*x*_ to a point.

### Reference pulse preparation

The reference pulse is obtained from the initial pulsed beam before entering the ST wave packet synthesis stage via a beam splitter BS_1_. The beam power is adjusted using a neutral density filter, and the spatial profile is enlarged by adding a spatial filtering system consisting of two lenses and a pinhole of diameter 30 μm. The spherical lenses are L_5−s_ of focal length *f* = 50 cm and L_6−s_ of focal length *f* = 10 cm, and they are arranged such that the pinhole lies at the Fourier plane. The spatially filtered pulsed reference then traverses an optical delay line before being brought together with the ST wave packet.

### Beam analysis

The ST wave packet is imaged from the plane of the grating G to an output plane via a telescope system comprising two cylindrical lenses L_2−*x*_ and L_3−*x*_ of focal lengths 40 cm and 10 cm, respectively, arranged in a 4*f* system. This system introduced a demagnification by a factor 4×, which modifies the spatial spectrum of the ST wave packet. The phase pattern displayed by the SLM is adjusted to pre-compensate for this modification. The ST wave packet and the reference pulse are then combined into a common path via a beam splitter BS_3_. A CCD camera (CCD_1_) records the interference pattern resulting from the overlap of the ST wave packet and reference pulse, which takes place only when the two pulses overlap also in time; see Supplementary Fig. 2.

### Group velocity measurements

Moving CCD_1_ a distance Δ*z* introduces an extra common distance in the path of both beams. However, since the ST wave packet travels at a group velocity *v*_g_ and the reference pulse at *c*, a relative group delay of $\Delta \tau = \Delta z({\textstyle{1 \over c}} - {\textstyle{1 \over {v_{\mathrm{g}}}}})$ is introduced and the interference at CCD_1_ is lost if ${\mathrm{\Delta }}\tau \gg {\mathrm{\Delta }}T$, where Δ*T* is the width of the ST wave packet in time. The delay line in the path of the reference pulse is then adjusted to introduce a delay *τ* = Δ*τ* to regain the interference. In the subluminal case *v*_g_ < *c*, the reference pulse advances beyond the ST wave packet, and the interference is regained by increasing the delay traversed by the reference pulse with respect to the original position of the delay line. In the superluminal case *v*_g_ > *c*, the ST wave packet advances beyond the reference pulse, and the interference is regained by reducing the delay traversed by the reference pulse with respect to the original position of the delay line. When *v*_g_ takes on negative values, the delay traversed by the reference pulse must be reduced even further. Of course, in the luminal case the visibility is not lost by introducing any extra common path distance Δ*z*. See Supplementary Fig. 3 for a graphical depiction.

From this, the group velocity is given by

16 $$v_{\mathrm{g}} = \frac{{{\mathrm{\Delta }}z}}{{{\mathrm{\Delta }}z/c - {\mathrm{\Delta }}\tau }}.$$

For a given value of Δ*z*, we fit the temporal profile *I*(0, 0, *τ*) to a Gaussian function to determine its center from which we estimate Δ*τ*. For each tilt angle *θ*, we repeat the measurement for three different values of Δ*z* and set one of the positions as the origin for the measurement set: 0 mm, 2 mm, and 4 mm in positive subluminal case (Supplementary Fig. 4a); 0 mm, 5 mm, and 10 mm in the positive superluminal case (Supplementary Fig. 4b); and 0 mm, −5 mm, and −10 mm in the negative-*v*_g_ case (Supplementary Fig. 4c). Finally, we fit the obtained values to a linear function, where the slope corresponds to the group velocity. The uncertainty in estimating the values of *v*_g_ (Δ*v*_g_ in Supplementary Table 1 and error bars for Fig. 4) are obtained from the standard error in the slope resulting from the linear regression.

## Supplementary information

Supplementary Information

## Data Availability

The data that support the findings of this study are available from the corresponding author upon reasonable request.

## References

[1] Brillouin L (1960). Wave Propagation and Group Velocity.

[2] Schulz-DuBois EO (1969). Energy transport velocity of electromagnetic propagation in dispersive media. Proc. IEEE.

[3] Boyd RW, Gauthier DJ (2009). Controlling the velocity of light pulses. Science.

[4] Hau LV, Harris SE, Dutton Z, Behroozi C (1999). Light speed reduction to 17 m per second in an ultracold atomic gas. Nature.

[5] Kash MM (1999). Ultraslow group velocity and enhanced nonlinear optical effects in a coherently driven hot atomic gas. Phys. Rev. Lett..

[6] Wang LJ, Kuzmich A, Dogariu A (2000). Gain-assisted superluminal light propagation. Nature.

[7] Song KY, Herráez MG, Thévenaz L (2005). Gain-assisted pulse advancement using single and double Brillouin gain peaks in optical fibers. Opt. Express.

[8] Casperson L, Yariv A (1971). Pulse propagation in a high-gain medium. Phys. Rev. Lett..

[9] Gehring GM, Schweinsberg A, Barsi C, Kostinski N, Boyd RW (2005). Observation of backward pulse propagation through a medium with a negative group velocity. Science.

[10] Baba T (2008). Slow light in photonic crystals. Nat. Photon..

[11] Dolling G, Enkrich C, Wegener M, Soukoulis CM, Linden S (2005). Simultaneous negative phase and group velocity of light in a metamaterial. Science.

[12] Steinberg AM, Kwiat PG, Chiao RY (1993). Measurement of the single-photon tunneling time. Phys. Rev. Lett..

[13] Tsakmakidis KL, Hess O, Boyd RW, Zhang X (2017). Ultraslow waves on the nanoscale. Science.

[14] Giovannini D (2015). Spatially structured photons that travel in free space slower than the speed of light. Science.

[15] Bouchard F, Harris J, Mand H, Boyd RW, Karimi E (2016). Observation of subluminal twisted light in vacuum. Optica.

[16] Lyons A (2018). How fast is a twisted photon?. Optica.

[17] Turunen J, Friberg AT (2010). Propagation-invariant optical fields. Prog. Opt..

[18] Hernández-Figueroa, H. E., Recami, E. & Zamboni-Rached, M (eds). *Non-Diffracting Waves* (Wiley-VCH, Weinheim, 2014)

[19] Donnelly R, Ziolkowski R (1993). Designing localized waves. Proc. R. Soc. Lond. A.

[20] Kondakci HE, Abouraddy AF (2017). Diffraction-free space-time beams. Nat. Photon..

[21] Longhi S (2004). Gaussian pulsed beams with arbitrary speed. Opt. Express.

[22] Saari P, Reivelt K (2004). Generation and classification of localized waves by Lorentz transformations in Fourier space. Phys. Rev. E.

[23] Saari P, Reivelt K (1997). Evidence of X-shaped propagation-invariant localized light waves. Phys. Rev. Lett..

[24] Alexeev I, Kim KY, Milchberg HM (2002). Measurement of the superluminal group velocity of an ultrashort Bessel beam pulse. Phys. Rev. Lett..

[25] Bonaretti F, Faccio D, Clerici M, Biegert J, Di Trapani P (2009). Spatiotemporal amplitude and phase retrieval of Bessel-X pulses using a Hartmann-Shack sensor. Opt. Express.

[26] Bowlan P (2009). Measuring the spatiotemporal field of ultrashort Bessel-X pulses. Opt. Lett..

[27] Lu JY, Greenleaf JF (1992). Nondiffracting X waves – exact solutions to free-space scalar wave equation and their finite aperture realizations. IEEE Trans. Ultrason. Ferroelec. Freq. Control.

[28] Di Trapani P (2003). Spontaneously generated X-shaped light bullets. Phys. Rev. Lett..

[29] Faccio D (2006). Conical emission, pulse splitting, and X-wave parametric amplification in nonlinear dynamics of ultrashort light pulses. Phys. Rev. Lett..

[30] Faccio D (2007). Spatio-temporal reshaping and X wave dynamics in optical filaments. Opt. Express.

[31] Dallaire M, McCarthy N, Piché M (2009). Spatiotemporal bessel beams: theory and experiments. Opt. Express.

[32] Jedrkiewicz O, Wang YD, Valiulis G, Di Trapani P (2013). One dimensional spatial localization of polychromatic stationary wave-packets in normally dispersive media. Opt. Express.

[33] Kuntz KB (2009). Spatial and temporal characterization of a bessel beam produced using a conical mirror. Phys. Rev. A.

[34] Lõhmus M (2012). Diffraction of ultrashort optical pulses from circularly symmetric binary phase gratings. Opt. Lett..

[35] Piksarv P (2012). Temporal focusing of ultrashort pulsed Bessel beams into Airy-Bessel light bullets. Opt. Express.

[36] Kondakci HE, Abouraddy AF (2016). Diffraction-free pulsed optical beams via space-time correlations. Opt. Express.

[37] Parker KJ, Alonso MA (2016). The longitudinal iso-phase condition and needle pulses. Opt. Express.

[38] Liu Z, Fan D (1998). Propagation of pulsed zeroth-order Bessel beams. J. Mod. Opt..

[39] Sheppard CJR (2002). Generalized Bessel pulse beams. J. Opt. Soc. Am. A.

[40] Zapata-Rodríguez CJ, Porras MA, Miret JJ (2008). Free-space delay lines and resonances with ultraslow pulsed Bessel beams. J. Opt. Soc. Am. A.

[41] Valtna H, Reivelt K, Saari P (2007). Methods for generating wideband localized waves of superluminal group velocity. Opt. Commun..

[42] Zapata-Rodríguez CJ, Porras MA (2006). X-wave bullets with negative group velocity in vacuum. Opt. Lett..

[43] Kondakci HE, Abouraddy AF (2018). Airy wavepackets accelerating in space-time. Phys. Rev. Lett..

[44] Bélanger PA (1986). Lorentz transformation of packetlike solutions of the homogeneous-wave equation. J. Opt. Soc. Am. A.

[45] Porras MA (2017). Gaussian beams diffracting in time. Opt. Lett..

[46] Porras MA (2018). Nature, diffraction-free propagation via space-time correlations, and nonlinear generation of time-diffracting light beams. Phys. Rev. A.

[47] Bhaduri B, Yessenov M, Abouraddy AF (2018). Meters-long propagation of diffraction-free space-time light sheets. Opt. Express.

[48] Kondakci HE (2018). Synthesizing broadband propagation-invariant space-time wave packets using transmissive phase plates. Opt. Express.

[49] Sainte-Marie A, Gobert O, Quéré F (2017). Controlling the velocity of ultrashort light pulses in vacuum through spatio-temporal couplings. Optica.

[50] Froula DH (2018). Spatiotemporal control of laser intensity. Nat. Photon..

[51] Averchi A (2008). Phase matching with pulsed Bessel beams for high-order harmonic generation. Phys. Rev. A.

[52] Bahabad A, Murnane MM, Kapteyn HC (2010). Quasi-phase-matching of momentum and energy in nonlinear optical processes. Nat. Photon..

[53] Turnbull D (2018). Raman amplification with a flying focus. Phys. Rev. Lett..

[54] Turnbull D (2018). Ionization waves of arbitrary velocity. Phys. Rev. Lett..

[55] Byrnes T, Kim NY, Yamamoto Y (2014). Exciton-polariton condensates. Nat. Phys..

