# Supplementary material for: Optical space-time wave packets having arbitrary group velocities in free space
Source: Nat Commun. 2019 Feb 25;10:929. doi: 10.1038/s41467-019-08735-8 (PMC6389885; doi:10.1038/s41467-019-08735-8)
Supplement: Supplementary file 1 — Supplementary Information [file 41467_2019_8735_MOESM1_ESM.pdf]

Optical space-time wave packets having arbitrary group velocities in free space:

Supplementary Material

Kondakci *et al*.

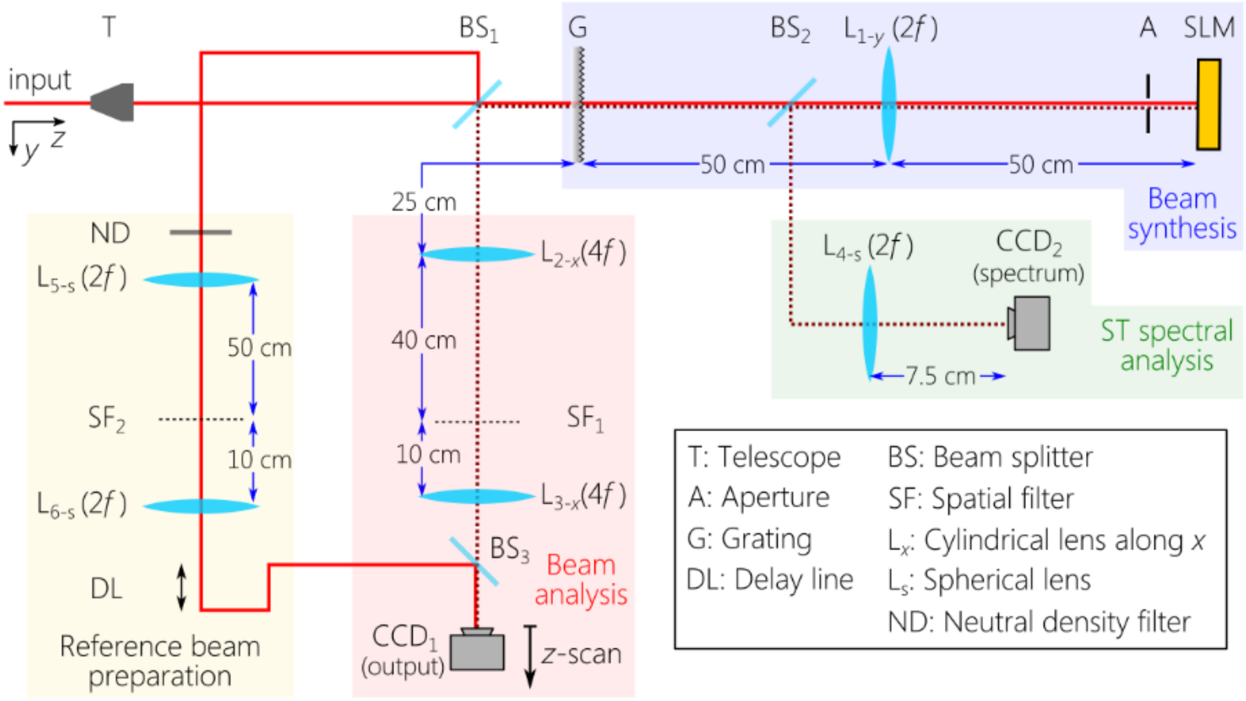

Supplementary Figure 1.

Detailed experimental setup. The experimental arrangement shown schematically in Fig. 2a in the main text is presented here in detail. The setup comprises four sections for space-time (ST) wave packet synthesis and characterization. The acronyms on all the optical components are provided in the inset box.

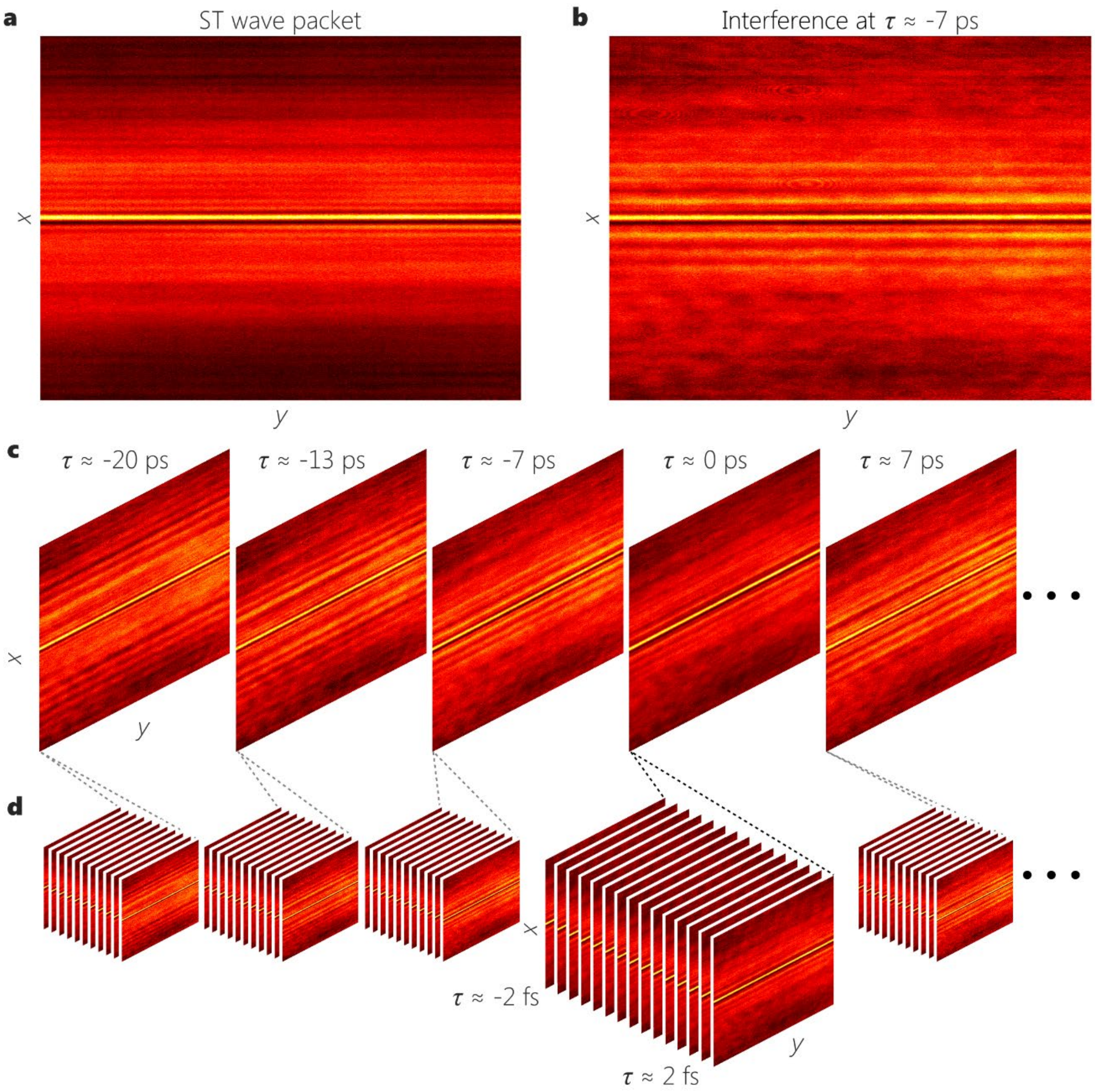

Supplementary Figure 2.

Restructuring the spatio-temporal wave packet profile from interferometric measurements. **a**, Time-integrated beam profile of a ST wave packet recorded by a slow detector (CCD_1) at a fixed propagation distance. In absence of the reference or if the reference and ST wave packet do not overlap in time, off-axis spatial interference along x is absent. **b**, Spatially-resolved interference fringes along the x-axis for a delay $\tau \approx 7$ ps, resulting from the overlap of the reference and ST wave packet in time. **c**, The visibility of the spatially resolved interference fringes changes as the delay is scanned around the center of the ST wave packet. **d**, A set of measurements with small delay increments are taken to obtain the visibility ν near the vicinity of any selected τ. Each set of such measurements yields a single line in the spatio-temporal profile $I(x, 0, \tau)$ given in Fig. 3b in the main text.

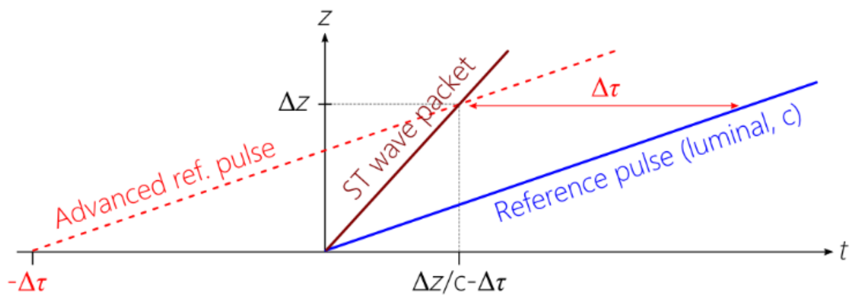

Supplementary Figure 3.

Space-time-diagram for determining the group velocity of ST wave packets. The experiment starts with the ST wave packet and reference pulse overlapping in space and time, resulting in high-visibility spatially resolved fringes (Supplementary Figure 2b). A common distance Δz is introduced into the path of the ST wave packet and the reference pulse by moving CCD_1 (Supplementary Figure 1), which results in a loss of the interference fringes (Supplementary Figure 2a). A delay $\Delta\tau$ is then introduced into the path of the reference pulse to regain the visibility of the spatial resolved interference fringes (Supplementary Figure 2c).

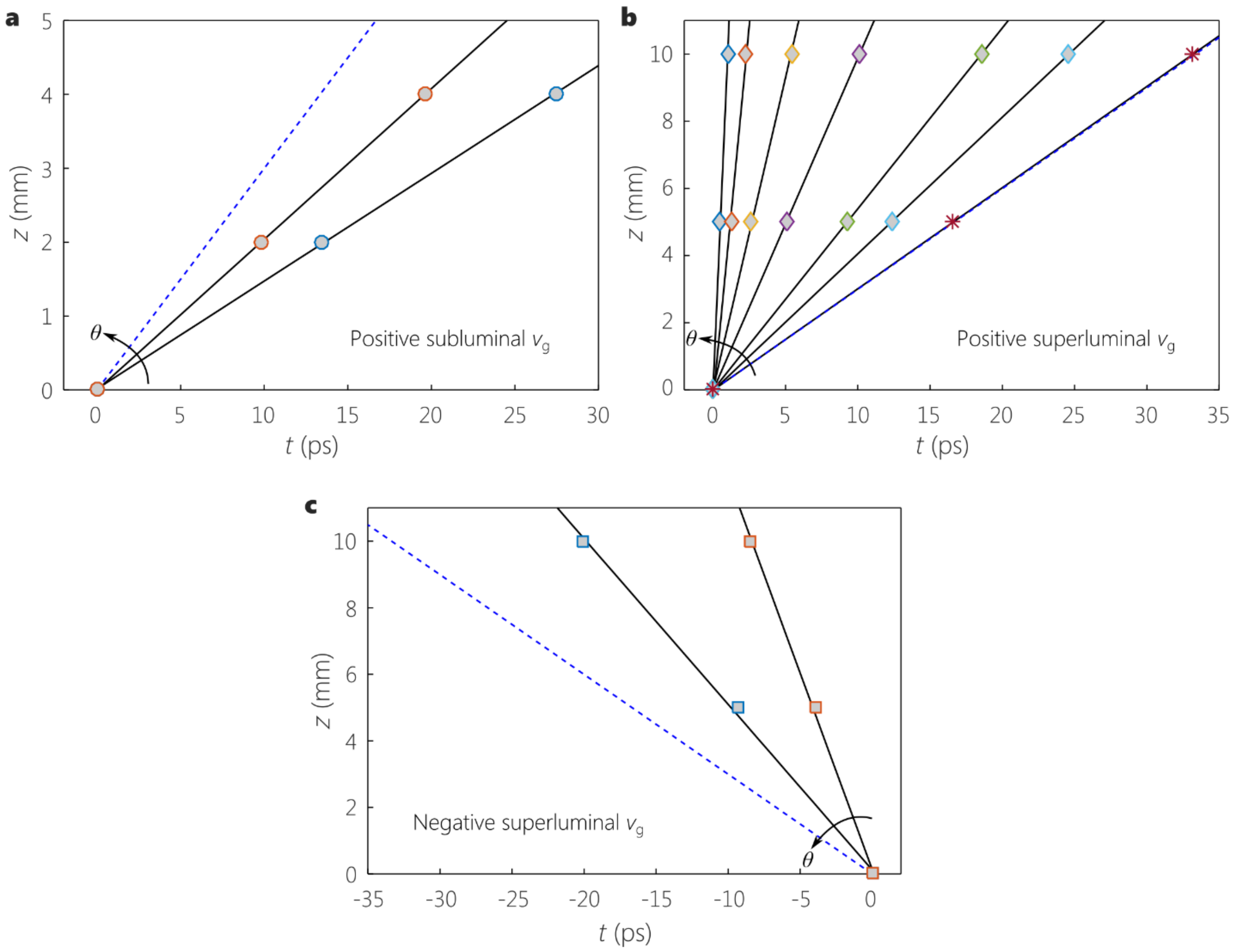

Supplementary Figure 4.

Space-time-diagram measurements of ST wave packets to estimate their group velocities. a, Positive subliminal ST wave packets lie below the light line $v_g = c$ (blue-dashed). A retardation of the reference pulse is required to obtain maximum visibility. **b**, Positive superluminal ST wave packets lie above the light line with a positive slope. An advancement of the reference pulse is required to obtain maximum visibility. The luminal ST wave packet, which coincides – as expected – with the light line (data points represented by stars) is produced by idling the SLM. **c**, Negative superluminal ST wave packets lie above the light line with a negative slope $v_g = -c$. An advancement of the reference pulse is required to obtain maximum visibility. Note that the required advancement here exceeds that of the case of positive superluminal ST wave packets. **a-c**, In all cases, three measurements are taken at three points in z and the required advancement or retardation of the reference pulse that gives the maximum visibility is recorded. One of the observation points set to be origin ($z = 0$). The black lines are linear fits.

Supplementary Table 1.

Measurement results and theoretical expectation for the group velocity of ST wave packets in free space

	$\boldsymbol{\theta}$	**Wave packet type**	$\boldsymbol{v_{\mathrm{g}}}$	$\boldsymbol{\Delta v_{\mathrm{g}}}$	**Theory**	**Conic section**
(1)	$26.6°$	Positive subluminal	$0.49c$	$\pm 3 \times 10^{-4}c$	$0.5c$	ellipse
(2)	$34.6°$	Postive subluminal	$0.68c$	$\pm 2 \times 10^{-5}c$	$0.69c$	ellipse
(3)	$45°$	Positive luminal	c	$\pm 3 \times 10^{-5}c$	c	line
(4)	$53.3°$	Positive superluminal	$1.36c$	$\pm 4 \times 10^{-4}c$	$1.34c$	hyperbola
(5)	$61.1°$	Positive superluminal	$1.80c$	$\pm 3 \times 10^{-4}c$	$1.81c$	hyperbola
(6)	$73.4°$	Positive superluminal	$3.29c$	$\pm 2 \times 10^{-4}c$	$3.36c$	hyperbola
(7)	$80.7°$	Positive superluminal	$6.17c$	$\pm 0.02c$	$6.14c$	hyperbola
(8)	$86.4°$	Positive superluminal	$14.86c$	$\pm 1.07c$	$15.9c$	hyperbola
(9)	$88.5°$	Positive superluminal	$32.86c$	$\pm 2.18c$	$39.21c$	hyperbola
(10)	$104.2°$	Negative superluminal	$-3.94c$	$\pm 0.03c$	$-3.94c$	hyperbola
(11)	$120.6°$	Negative superluminal	$-1.66c$	$\pm 5 \times 10^{-3}c$	$-1.69c$	Hyperbola

The group velocity v_{g} of the ST wave packets are arranged in order of increasing value of θ in the range $0 < \theta < 180°$. The theoretical values correspond to $v_{\mathrm{g}} = \tan\theta$, and Δv_{g} is the uncertainty in the measured value of v_{g}.
